# PROTOCOL: Effectiveness of road safety interventions: An evidence and gap map

**DOI:** 10.1002/cl2.1077

**Published:** 2020-03-02

**Authors:** Dinesh Mohan, Geetam Tiwari, Mathew Varghese, Kavi Bhalla, Denny John, Ashrita Saran, Howard White

**Affiliations:** ^1^ TRIPP, Indian Institute of Technology New Delhi India; ^2^ Head Orthopaedics Department St. Stephen's Hospital New Delhi India; ^3^ Department of Public Health Sciences University of Chicago Chicago Illinois; ^4^ Campbell South Asia, ISID Complex New Delhi India; ^5^ Campbell Collaboration New Delhi India

## Abstract

The Decade of Action for Road Safety 2011–2020, officially proclaimed by the UN General Assembly in March 2010, sought to reduce morbidity and mortality due to road traffic injuries (RTI) significantly. While there is reasonable agreement internationally on safer designs of motor vehicles (except locally produced vehicles like three‐wheeled scooter taxis, tuk‐tuks, jeepneys, etc.), there is a lack of evidence based interventions in road and infrastructure design, police enforcement and post‐crash care. Researchers in the field of traffic safety have been aware of the existence of counterintuitive results in their area of work for more than four decades. The fact that many interventions do not result in reductions in RTI is mainly because a large number of studies only measure intermediate outcomes like change in behaviour or knowledge and not the actual results in the field. The scope of this evidence and gap map (EGM) is to cover relevant studies in road safety sector from all countries and present the effectiveness of interventions in terms of mainly traffic crash injuries as its outcome. The interventions adopted in this EGM are classified into five broad categories: Human factors, vehicle factors and protective devices, road design, infrastructure and traffic control, post‐crash pre‐hospital care and legal and institutional framework. In order to come closer to accomplishing targets for road safety, it is important to allocate resources to promote interventions that are effective in achieving outcomes in the context of road safety. A mapping will provide a comprehensive overview of existing knowledge in the area of road safety and its effectiveness across the world. The map will guide programme managers to high quality evidence and inform targeted commissioning of future research.

## 1 BACKGROUND

### 1.1 The problem

The World Health Organization (WHO) released its World Report on Road Traffic Injury Prevention in 2004 (Peden et al., 2004). This report focused on road traffic injuries (RTI) and fatalities as a worldwide health problem and included a summary of the known risk factors associated with road traffic crashes and possible countermeasures that should be put in place to control the problem. It also pointed out that without new or improved interventions, RTI will be the third leading cause of death by the year 2020. The publication of this report spurred some national and international agencies and civil society groups to give more attention to the problem of road safety and a number of resolutions have been passed by the United Nations General Assembly, World Health Assembly and the Executive Board of the WHO (WHO, 2004, 2009, 2011, 2016).

As a follow up, two Global Ministerial Conferences on Road Safety have been held in Moscow, Russia (19–20 November 2009), and Brasilia, Brazil (18–19 November 2015). And the third one is scheduled in February 2010 in Stockholm, Sweden. At the close of the conference in Brasilia, the 2,200 delegates adopted the Brasilia Declaration on Road Safety through which they agreed ways to halve road traffic deaths by the end of this decade—a key milestone within the new Sustainable Development Goal (SDG) target 3.6 (Brasilia Declaration, 2015). This target has not been achieved and the total number of RTI and fatalities are still increasing in most countries of the world.

The WHO has also released four Global Status Reports on Road Safety in 2009, 2013, 2015 and 2018. The final one being the Global Status Report on Road Safety 2018 (WHO, 2018). These reports give a broad assessment of the status of road safety in ~180 countries. The data were obtained from national governments using a standardized survey form. The GSRRS18 shows that the overall global road traffic fatality rate is 17.4 per 100,000, but there is a great disparity by income and regions. Low‐ and middle‐income countries (LMIC) are reported to have the highest annual road traffic fatality rates, at 24.1 per 100 000, while the rate in high‐income countries (HIC) is lowest at 9.2 per 100,000, and that over half of those who die in road traffic crashes are pedestrians, bicyclists and users of motorized two‐wheelers (MTW).

In LMIC traffic injuries have been steadily rising, and now rank in the top 10 causes of death (WHO, 2018). Over 50% of the victims are pedestrians, bicyclists and motorcyclists and RTI has become the leading cause of death for young adults in most countries. While most HIC have well‐established road safety policies, many LMICs are in the process of establishing national regulatory agencies and sustainable funding streams to support large scale interventions that systematically address risky behaviours and the safety characteristics of vehicle and road infrastructure.

LMIC are also growing their rural road and highway infrastructure. National governments and international development agencies consider the expansion of road infrastructure a key strategy for economic and social development. In the last two decades, China has built a highway system that rivals that of the United States, with plans of substantial expansion (Xu & Nakajima, 2017; Yan, 2011). In India, the rapid growth of the highway infrastructure is currently underway because insufficient road transport is viewed as a key impediment to industrial growth (Ghani, Goswami, & Kerr, 2013). Africa, where most people do not have access to all‐weather roads, plans to expand its road network by 6–10 times by 2040 (Programme for Infrastructure Development in Africa, 2013). This increase in road infrastructure and number of vehicles is likely to result in an increase in RTI rates unless accompanied by appropriate evidence‐based road safety interventions.

The Decade of Action for Road Safety 2011–2020, officially proclaimed by the UN General Assembly in March 2010, seeks to save millions of lives by building road safety management capacity; improving the safety of road infrastructure; further developing the safety of vehicles; enhancing the behaviour of road users; and improving post‐crash response. Several national and international initiatives have been taken over the past decade to promote and fund road safety initiatives around the world. While there is reasonable agreement internationally on safer designs of motor vehicles (except locally produced vehicles like three‐wheeled scooter taxis, tuk‐tuks, jeepneys, etc.), there is a lack of evidence‐based interventions in road and infrastructure design, police enforcement and post‐crash care (Davey & Freeman, 2011; Hauer, 2019; Wilson & Gangathimmaiah, 2017).

### 1.2 Impact of RTI on society

A very large number of high income countries have been estimating the costs of road traffic crashes over the past three decades. The methods used and costs allocated have generated a great deal of discussion and debate, in particular, because of the difficulty of putting monetary values on pain and suffering. A study undertaken by the European Federation of Road Traffic Victims on the impact of road death and injury in collaboration with the Commission for European Union gives the following qualitative conclusions regarding the effect of road traffic crashes on victims (Haegi, 1995).

- Physical and mental impairment through road traffic injury can have long‐term effects which deny victims the ability to maintain their standard of living.
- A large proportion of the relatives of dead and disabled victims, as well as the disabled themselves, suffer psychological disorders. The worst situation is that of the relatives of the dead.
- The bereaved are the worst affected—70%—by relationship problems, communication difficulties and sexual problems. The figure for relatives of disabled victims is 40%, and for the disabled themselves 50%. After 3 years these problems do not decrease as one would expect, but worsen for each category by about 5 points.
- About 50% of the relatives of victims, and the victims themselves, state that for extended periods they consume more psychotropic products like tranquillizers, sleeping tablets, tobacco, alcohol and drugs than before the incident.
- It is sometimes believed that due to the tragedy, the relationship of the respondents with their normal social partners deteriorates.
- The capacity to enjoy life as before the crash tragically disappears for 91% of the relatives of dead victims for the first 3 years. After this period, the loss persists for long periods for 84% of them. For many, this loss will be permanent.

We have quoted from this report extensively because it is important to note that economic costing of human tragedies can only be used as an inefficient tool to understand the partial costs of the problem.

### 1.3 Scope of the evidence and gap map (EGM)

Road safety interventions are described as the interaction of three components in the light of Haddon's matrix (Haddon, 1968): human, vehicle and environment separated into pre‐crash, crash and post‐crash phases. This has been strengthened further over the past two decades by focussing on the Safe Systems Approach which also includes strengthening of institutions and other road safety policy measures (Bliss & Breen, 2009) The interventions included in the EGM here will focus mainly on their impact on road traffic‐related injuries as its primary outcome and selected safe road‐use practises as its intermediate outcome. In the late 1960s and early 1970s, there was a significant paradigm shift in the development of countermeasures to reduce motor vehicle crash injuries and deaths. Prior to this time countermeasures were largely based on folklore and were not evaluated; the new paradigm envisaged a much broader range of countermeasures and emphasized the importance of evaluating them. There are several modes of transport which are widely used in LMICs such as motorcyclists, three‐wheelers, but very limited literature is available on effectiveness of various interventions on road safety with heterogeneous composition of traffic. The EGM will cover all aspects of road safety interventions except car‐design. Safety standards for car design including crash worthiness standards have been evolving since 1970s. The UNECE WP.29 (United Nations Economic Commission for Europe, 2012) vehicle safety standards and New Car Assessment Programme (NCAP; Hobbs and McDonough, 1998) are also attempting to harmonize vehicle standards internationally. There is general consensus on technologies which work and don't work internationally and car designs around the world are converging to similar international standards. Safer car designs are also influenced by the market because of car safety ratings announced by agencies like NCAP. However, infrastructure design, safety policies and enforcement are not subject to market mechanisms in the same way. Hence, car design interventions have been excluded from EGM.

The scope of this EGM is to cover relevant studies in the road safety sector from all countries and present the effectiveness of interventions in terms of mainly traffic crash injuries as its outcome.

The interventions adopted in this EGM are classified into five broad categories: Human factors, vehicle factors and protective devices, road design, infrastructure and traffic control, post‐crash pre‐hospital care and legal and institutional framework. They are described as follows:

- Human factors: These cover all interventions including any factor or road user behaviour that leads to occurrence or consequence of RTI.
- Vehicle factors and protective devices: These are mainly focused on design of different vehicle modes except cars and protective equipment which may lead to reduction in injuries.
- Road design, infrastructure and traffic control: These interventions cover various types of infrastructure (geometry, traffic control, etc.) present on different categories of roads (urban and rural roads) and are critical factors affecting road traffic injury.
- Post‐crash pre‐hospital care: These pre‐hospital interventions (e.g., road side, in ambulance etc.) aims to reduce the severity of injury consequences once a road traffic crash has occurred.
- Legal and institutional framework: These mainly focus on insurance policies, vehicle taxes, fuel and road pricing, central government, research institutions and laws addressing road traffic injury.

### 1.4 Conceptual framework of the EGM

**Figure 1:**
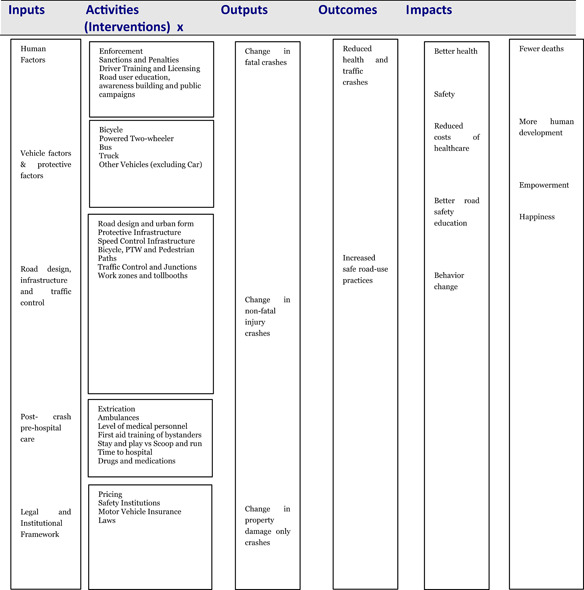
Conceptual framework for the EGM. *Source*: Authors based on White and Gunnarson (2008)

The conceptual framework links road safety interventions with the outcomes and impacts along the causal chain (Figure 1). The conceptual framework shows the causal chain through which the inputs are turned into final societal impacts, through activities, outputs and outcomes.

It is to be noted that the links in the causal chain are not automatic. For example, the use of police enforcement to reduce road user risk and behaviour would also need legal and institutional framework to reduce health and traffic crashes in order to reduce injuries and fatalities, thus improving health and resulting in less morbidity and mortality.

A scientific approach for controlling RTI evolved in HIC over the last 50 years or so. The main concept behind the evolving strategies rested on the understanding that we must move away from finding fault with victims and instituting retribution systems to a more reasoned approach that deals with systemic improvements and finding solutions which by and large do not put an extra burden on road users. It was understood that RTI result from a complex interaction of sociological, psychological, physical and technological phenomena and since injuries result from an exchange of energy between the environment and the human body, it is possible to develop safety policies and strategies in a scientific and comprehensive manner (Bliss & Breen, 2009; Haddon, 1980; Peden et al., 2004). This will require a shift to an approach with a result focus which aims to eliminate road deaths and serious injuries by adopting interventions that are more likely to succeed in aiming for Vision Zero (Svensson, 2018).

The intervention categories in this EGM are adapted from the Haddon's Matrix which presents the typology of road traffic crashes in time and space (Haddon, 1972). The time category is divided into three mutually exclusive categories comprising interventions that prevent the occurrence of a crash, prevent or reduce the severity of injuries during a crash, and reduce the possibility of a negative outcome after the crash. The space categories include those interventions that are associated with human beings, vehicles and rest of the environment. The last category includes the road infrastructure, legislation, policing and role of institutions. The systems approach as detailed by Bliss and Breen (2009) further elaborates the role of different institutions and inputs included in the environment category in Haddon's Matrix. For the purposes of the EGM these interventions have been clubbed into five groups: (a) human factors, (b) vehicle factors and protective devices, (c) road design, infrastructure and traffic control, (d) post‐crash pre‐hospital care, and (e) legal and institutional framework, and associated sub‐categories.

One of the reasons that road safety interventions were not particularly successful in the first 60 years of motorization (1910–1970) in any part of the world was because policy makers and researchers could not measure the effectiveness of various interventions in terms of fatalities, injuries or crashes prevented. Subsequent research indicated that some interventions may have been based on theoretical beliefs of their effectiveness but were not so in real use and others may produce behaviour change but not found to be effective in the real world of traffic. For example, recent systematic reviews indicate that the following interventions do not result in reducing the adverse effect of road traffic crashes:

- Advanced training in trauma life support for ambulance crews (Jayaraman, Sethi, & Wong, 2014).
- Interventions in the alcohol server setting for preventing injuries (Ker & Chinnock, 2008).
- Spinal immobilization for trauma patients (Kwan, Bunn, & Roberts, 2004).
- Motorcycle rider training for the prevention of road traffic crashes (Kardamanidis, Martiniuk, Ivers, Stevenson, & Thistlethwaite, 2010).
- Post‐licence driver education for the prevention of road traffic crashes (Ker et al., 2003).
- Safety education of pedestrians for injury prevention (Duperrex, Roberts, & Bunn, 2002).
- School‐based driver education for the prevention of traffic crashes (Roberts & Kwan, 2003).

Researchers in the field of traffic safety have been aware of the existence of counterintuitive results in their area of work more than four decades. The fact that many interventions do not result in reductions in RTI is mainly because a large number of studies only measure intermediate outcomes like change in behaviour or knowledge and not the actual results in the field. For example, Duperrex et al. (2002) point out that “Pedestrian safety education can result in improvement in children's knowledge and can change observed road crossing behaviour, but whether this reduces the risk of pedestrian motor vehicle collision and injury occurrence is unknown”. Hauer (2019) goes further and states that “Issues of concern are the lightness with which decisions affecting road‐user safety can be based on opinion that is unsupported by evidence…that questionable results can be given a ring of consensual truth, and that the questions which research asks and what findings get published are at times influenced by external interest”.

In this EGM importance has been given to primary outcomes as there is little agreement among researchers on the relationship between behavioural/knowledge changes and reductions in traffic crashes. Therefore, studies are included only if the outcome is measured in changes in RTI (fatal, injury only) or use of protective devices, changes in vehicle speeds or drinking and driving.

### 1.5 Why it is important to develop the EGM

In a recent paper Hauer (2019) states:

Over the past two decades or so progress has been made towards evidence‐based practice. Research and researchers provided valuable tools for practitioners to use. But much of practice is still opinion‐based and the role of research remains ambiguous. The first step towards reform is to rethink and then to revamp the research‐practice relationship. The reformed relationship should be endowed with a purposeful structure, one that cures what dysfunction there is and promotes the generation of trustworthy evidence.

Unfortunately, in the absence of local research on road‐related safety interventions, roads and highways in LMIC are being designed to safety standards of HIC without an adequate understanding of the evidence base of existing standards. For example, it is generally accepted that traffic calming measures like chicanes, road narrowing and roundabouts are effective in reducing RTI (Bunn et al., 2003). However, effectiveness of some of these measures in LMICs is not known as vehicles like MTW may not be affected. Even in HIC, many standards for vehicles, roads and policing activities are being promoted without the availability of adequate scientific evidence regarding their effectiveness (Elvik, 2017; Hauer, 2019). Many systematic reviews also point out the fact that evidence for road safety interventions may be available for HICs, but the same is lacking from LMICs (Mulvaney et al., 2015; Roberts & Kwan, 2003).

The SDG target 3.6 for road safety aims to halve the number of global deaths and injuries from road traffic accidents by 2020. However, considering the example of WHO European region, despite a decrease of 8.1% in road traffic deaths between 2010 and 2013, the region may not be able to fulfil this SDG 2020 target to halve the road traffic fatalities (Jackisch, Sethi, Mitis, Szymañski, & Arra, 2015; WHO, 2018)

The SDG target 11.2 seeks to provide access to safe, affordable, accessible and sustainable transport systems for all, improving road safety, notably by expanding public transport, with special attention to the needs of those in vulnerable situations, women, children, people with disabilities and older people.

In order to come closer to accomplishing these targets, it is important to allocate resources to promote interventions that are effective in achieving outcomes in the context of road safety. A mapping will provide a comprehensive overview of existing knowledge in the area of road safety and its effectiveness across the world. The map will guide programme managers to high quality evidence and inform targeted commissioning of future research.

### 1.6 Existing EGMs and relevant systematic reviews

A map of evidence maps conducted in LMIC identified no EGM conducted around transportation and other adaptive measures (Phillips et al., 2017). This EGM being a global focus aims to include studies on the effectiveness of road safety interventions from countries in all continents. The EGM strives to capture relevant studies conducted and broaden the included interventions and outcomes to better reflect the state of evidence in road safety in 2019.

The following systematic reviews and synthesis studies will be incorporated in the EGM after being subjected to inclusion/exclusion criteria.

#### 1.6.1 Cochrane reviews

Beyer, F. R., & Ker, K. (2009). Street lighting for preventing road traffic injuries. *Cochrane Database of Systematic Reviews*, Issue 1, Art. No.: CD004728. https://doi.org/10.1002/14651858.CD004728.pub2

Bunn, F., Collier, T., Frost, C., Ker, K., Steinbach, R., Roberts, I., & Wentz, R. (2003). Area‐wide traffic calming for preventing traffic related injuries. *Cochrane Database of Systematic Reviews*, Issue 1, Art. No.: CD003110. https://doi.org/10.1002/14651858.CD003110

Desapriya, E., Harjee, R., Brubacher, J., Chan, H., Hewapathirane, D. S., & Subzwari, S., Pike, I. (2014). Vision screening of older drivers for preventing road traffic injuries and fatalities. *Cochrane Database of Systematic Reviews 2014*, Issue 2, Art. No.: CD006252. https://doi.org/10.1002/14651858.CD006252.pub4

Duperrex, O., Roberts, I., & Bunn, F. (2002). Safety education of pedestrians for injury prevention. *Cochrane Database of Systematic Reviews*, Issue 2, Art. No.: CD001531. https://doi.org/10.1002/14651858.CD001531

Galvagno, S. M. Jr., Sikorski, R., Hirshon, J. M., Floccare, D., Stephens, C., Beecher, D., & Thomas, S. (2015). Helicopter emergency medical services for adults with major trauma. Cochrane Database of Systematic Reviews, Issue 12, Art. No.: CD009228. https://doi.org/10.1002/14651858.CD009228.pub3

Kwan, I., & Mapstone, J. (2006). Interventions for increasing pedestrian and cyclist visibility for the prevention of death and injuries. *Cochrane Database of Systematic Reviews*, Issue 4, Art. No.: CD003438. https://doi.org/10.1002/14651858.CD003438.pub2

Liu, B. C., Ivers, R., Norton, R., Boufous, S., Blows, S., Lo, S. K. (2008). Helmets for preventing injury in motorcycle riders. *Cochrane Database of Systematic Reviews*, Issue 1, Art. No.: CD004333. https://doi.org/10.1002/14651858.CD004333.pub3

Macpherson, A., & Spinks, A. (2008). Bicycle helmet legislation for the uptake of helmet use and prevention of head injuries. *Cochrane Database of Systematic Reviews*, Issue 3, Art. No.: CD005401. https://doi.org/10.1002/14651858.CD005401.pub3

Martin, A. J., Marottoli, R., O'Neill, D., Martin, A. J., Marottoli, R., & O'Neill, D. (2013). Driving assessment for maintaining mobility and safety in drivers with dementia. *Cochrane Database of Systematic Reviews*, Issue 8, Art. No.: CD006222. https://doi.org/10.1002/14651858.CD006222.pub4

Mulvaney, C. A., Smith, S., Watson, M. C., Parkin, J., Coupland, C., Miller, P., Kendrick, D., & McClintock, H. (2015). Cycling infrastructure for reducing cycling injuries in cyclists. *Cochrane Database of Systematic Reviews*, Issue 12, Art. No.: CD010415. https://doi.org/10.1002/14651858.CD010415.pub2

Wilson, C., Willis, C., Hendrikz, J. K., Le Brocque, R., & Bellamy, N. (2010). Speed cameras for the prevention of road traffic injuries and deaths. Cochrane Database of Systematic Reviews, Issue 11, Art. No.: CD004607. https://doi.org/10.1002/14651858.CD004607.pub4

#### 1.6.2 Non‐Cochrane reviews

Brieger, F., Hagen, R., Vetter, D., Dormann, C.F., & Storch, I. (2016). Effectiveness of light‐reflecting devices: A systematic reanalysis of animal‐vehicle collision data. *Accident & Prevention*, 97, 242–260.

Decker, J. S., Stannard, S. J., McManus, B., Wittig, S. M. O., Sisiopiku, V. P., & Stavrinos, D. (2015). The impact of billboards on driver visual behaviour: A systematic literature review. *Traffic Injury Prevention*, 16 (3).

Desapriya, E. Subzwari, S., Sasges, D., Basic, A., Alidina, A., Turcotte, K., & Pike, I. (2010). Do light truck vehicles (LTV) impose greater risk of pedestrian injury than passenger cars? A meta‐analysis and systematic review. *Traffic Injury Prevention*, 11 (1).

Elvik, R., & Greibe, P. (2005). Road safety effects of porous asphalt: A systematic review of evaluation studies. *Accident Analysis & Prevention*, 37, 515–522.

Fu, C., Zhang, Y., Qi, W., & Cheng, S. (2016). Effects of digital countdown timer on intersection safety & efficiency: A systematic review. *Traffic Injury Prevention*, 17 (1).

Huemer, AK., Schumacher, M., Mennecke M., & Vollrath, M. (2018). Systematic review of observational studies on secondary task engagement while driving. *Accident Analysis & Prevention*, 119, 225–236.

Kim, Chang‐Yeon., Wiznia.D.H., Averbukh, L., Dai, F., & Leslie, M.P. (2015). The economic impact of helmet use on motorcycle accidents: A systematic review and meta‐analysis of the literature from the past 20 years. *Traffic Injury Prevention*, 16 (7).

Napolitano, L. M. (2017). Prehospital tranexamic acid: What is the current evidence? *Trauma Surgery & Acute Care Open*, 2:e000056. https://doi.org/10.1136/tsaco‐2016‐000056

Roshandel, S., Zheng, Z, & Washington, S. (2015). Impact of real‐time traffic characteristics on freeway crash occurrence: Systematic review and meta‐analysis. *Accident Analysis & Prevention*, 79, 198–211.

Theofilatos, A., & Yannis, G. (2014). A review of the effect of traffic and weather characteristics on road safety. *Accident Analysis & Prevention*, 72, 244–256.

Unsworth, C.A., & Baker, A. (2014). Driver rehabilitation: A systematic review of the types and effectiveness of interventions used by occupational therapists to improve on‐road fitness‐to‐drive. *Accident Analysis & Prevention*, 71, 106–114.

#### 1.6.3 Synthesis of studies

The SafetyCube project^1^ synthesized existing knowledge on road safety risk factors and countermeasures in comprehensive synopses. These are listed as per risk factor/measure, colour code (assigned to reflect the strength of evidence on the effect of the risk factor or measure), and the road safety area concerned (behaviour, infrastructure, vehicle).

## 2 OVERALL AIM AND OBJECTIVES

The overall aim of the EGM is to gather and present any research on interventions aiming to reduce the RTI and fatalities any where in the world. This protocol provides a project plan to introduce the first EGM that takes into account the existing studies and newly published literature on the effectiveness of interventions pertaining to road safety. No comprehensive mapping has been done to show whether studies on road safety originate from LMIC or HIC. This EGM moves toward extending the scope of mapping such studies to a global scale.

The aim of the EGM is to identify, map, and describe the existing evidence on the effectiveness of interventions to improve road safety across all countries.

The objectives of this EGM are:

(1) to identify existing evidence from all effectiveness studies and systematic reviews (SRs) related to road safety interventions; and
(2) to identify existing gaps in evidence where new primary studies and systematic reviews could add value.

The EGM aims to direct the future research and discussions based on systematic evidence towards the approaches and interventions which are most effective in the road safety sector. This could enable generation of evidence for informing policy at global, regional or national levels.

## 3 METHODOLOGY

### 3.1 Defining EGMs

An EGM aims to provide a visual overview of thematic collections of what we know and do not know about the effectiveness of interventions (Snilstveit, Vojtkova, Bhavsar, Stevenson, & Gaarder, 2016).^2^. The interventions and outcomes are graphically presented in row lists and column lists respectively in the map, indicating the density and scarcity of the available evidence, and gives the confidence rating for systematic reviews. New primary studies fill the absolute gaps, while the new meta‐analyses and systematic reviews fill the synthesis gaps presented in the EGM. They are used as a global tool which attempts to give access of research evidence to practitioners, policy makers, the public, and research commissioners.

### 3.2 EGM framework

The framework for this EGM was constructed through a review of strategy and policy documents and discussion through external consultations:

1. An Advisory Group specially nominated for this project
2. Discussion with ICoRSI^3^ Council Members.
3. Reference to reviews/meta‐analysis in The Handbook of Road Safety Measures and Safety Cube Decision support system.

The identification of primary studies and systematic reviews to be included will be through a comprehensive search of previously published and unpublished literature irrespective of whether the study is completed or ongoing. This, in turn, will also help identify and hence fill the existing gaps in the evidence map.

The final EGM will have a structured framework of interventions and outcomes relevant to road safety with various filters in a user‐friendly way.

Key features include:

1. The EGM will contain all the relevant evidence from SRs and primary studies and provide access to user‐friendly summaries and appraisals of those studies.
2. The EGM will show where completed and, through the inclusion of trial registries, on‐going primary studies have been conducted.
3. The EGM will highlight absolute gaps (lack of studies for particular interventions/outcomes) and synthesis gaps (where there is a density of primary studies but lack of high‐quality SRs or an update of existing SRs)

#### 3.2.1 Intervention

The EGM will include any intervention aiming to reduce RTI and fatalities as an objective excluding the effectiveness of car design intervention in the vehicle factors and protective devise category. All the interventions possible in road safety have been categorized in groups and sub‐groups as shown in Table 1.

**Table 1:** Intervention classification

Intervention categories	Sub‐categories	Sub‐category explanation	Examples
1. Human factors	1.1 Enforcement	Actions taken to ensure compliance with legislation	(a) Speed and red‐light enforcement‐ Manual policing Speed cameras: Cameras that are positioned at a specific location to take pictures of vehicles exceeding speed limits Red light Camera: Cameras that automatically detect and record red light running vehicles at intersections (b) Drink‐driving/drug enforcement: Driving with alcohol or drugs in the blood stream (c) Seat‐belt enforcement: Enforcement of legislation requiring the fitting of seat belts to motor vehicles and the wearing of seat belts by motor vehicle occupants to be mandatory (d) Helmet enforcement: A helmet is a form of protective gear worn to protect the head during an impact. The intervention includes enforcement of laws for wearing helmets while driving (e) Cell phone use: Mobile phone use while driving (f) Other offence enforcement: Enforcement of laws preventing offensive driving, for example, reckless driving (g) Vehicle inspection: Procedure mandated by national or subnational governments, in which a vehicle is inspected to ensure that it conforms to regulations governing safety
	1.2 Sanctions and penalties	These are a means of enforcement for obedience of road safety laws and regulations	(a) Fines: A fine imposed for disobedience with the driving law or with rules and regulations aimed to reduce RTI (b) Imprisonment: It is a stringent form of sanction for disobedience with the driving laws or with rules and regulations (c) License demerit points: The system in which a driver's licensing authority, police force, or other organization issues cumulative demerits, or points to drivers on conviction for road traffic offenses. Points may either be added or subtracted, depending on the particular system in use. (d) License suspension: Temporary withdrawal of the right to drive a motor vehicle (e) Re‐education/Retraining programme: The programme in which drivers found committing any driving offence are required to take a retraining programme which may also be an alternative to prosecution
	1.3 Driver training and licensing		(a) Driver education and training: Training drivers for skill acquisition, decision making while driving or risk mitigation in context of reducing incidence of RTI. (b) Driver license age: The age at which a person may obtain a driver's license to lawfully drive a motor vehicle on public roads. (c) Driving test: The tests to evaluate person's knowledge on driving related rules and laws and to assess the person's ability to drive (hazard perception, Vision tests, health requirements) (d) Graduated driving licenses: The system designed to provide to provide novice drivers of motor vehicles with driving experience and skills gradually over time in low‐risk environments. (e) Screening by employer (driving record, health, star rating)
	1.4 Road user education, awareness building and public campaigns	Any educational programme or training that aims to teach appropriate skills of road safety to reduce RTI	a) General, road use (children, adults, all) b) Alcohol use c) Road signs d) Helmet use, Seatbelt use
2. Vehicle factors and protective devices	2.1 Bicycle	Bicycle is a human‐powered, pedal‐driven, single‐track vehicle, having two wheels attached to a frame, one behind the other.	(a) ABS (Anti‐lock braking system) and combined brakes, Emergency braking, speed limiters: The systems designed to prevent the problems occurring when wheels lock (b) Daytime running lights: Automotive lighting and bicycle lighting device on the front of a motor vehicle or bicycle, automatically switched on when the vehicle is in drive. (c) Reflective material: clothing worn that has highly reflective properties or a colour that is easily discernible from any background (d) Protective clothing: Clothing designed to mitigate risk of injury when rider is exposed to injury through contact with other objects (e) Helmets: Devices that reduces injuries by providing additional impact and abrasion protection to the head of a wearer in the event of a crash (f) Under‐run guards: The Under‐run protection bar enables small and large vehicles to make bumper‐to‐bumper contact thus preventing the smaller and lower vehicles to "ride under" the large vehicles especially trucks (g) Active protection: Autonomous Emergency Braking AEB, Electronic Stability Control (ESC) (h) Pedestrian protection: The use of vehicle design concepts that reduce the likelihood of injuries to pedestrians in the event of a car‐pedestrian crash. (i) Vehicle Design other than cars
	2.2 Powered two‐wheeler	A two‐wheeled vehicle powered by an engine	
	2.3 Bus	Bus is a motor vehicle designed to carry many passengers	
	2.4 Truck	Truck is a motor vehicle designed to transport cargo	
	2.5 Other vehicles (excluding car)	For example—three wheelers, electric vehicles, etc.	
3. Road design, infrastructure and traffic control	3.1 Road design and urban form		(a) Lane width, number of lanes (b) Shoulder: Designing the surface immediately beyond the carriageway edge line (c) Median: Design of the physical separation between opposing traffic streams (d) Road lighting: The application of illumination systems along roadways, primarily for the purpose of improving safety by increasing visibility of roadside hazards and by reducing the effects of glare from other light sources in the visual environment, such as vehicle headlamps. (e) Black spot treatment: A systematic and scientific identification of abnormally high accident sites or hazardous road locations and a remedial process that aims to develop appropriate and cost‐effective treatments for such sites. (f) Urban form: The physical patterns, layouts, and structures that make up an urban centre are collectively called the urban form
	3.2 Protective infrastructure		(a) Guard rails: Design of longitudinal highway barriers designed to reduce the impact of Run‐off‐road collisions (b) Crash cushions: A device intended to reduce the damage to structures, vehicles, and motorists resulting from a motor vehicle collision. These are designed to absorb the colliding vehicle's kinetic energy. (c) Roadside safety treatment: This aims to remove particularly dangerous and sight‐reducing obstacles from the roadside and give drivers greater opportunity to regain control in the event of run‐off the road, for example, flattening side slopes, increasing distance between edge and fixed obstacles and removal of such obstacles
	3.3 Speed control infrastructure		(a) Speed limits: Road speed limits are maximum speed permitted by legislation on a given stretch of road. They are indicated on a traffic sign (b) Traffic calming: Measures taken to reduce traffic volume and/or speed (e.g. bumps, humps, other raised pavement areas, street closures, traffic diversion etc.) (c) Speed reducing devices: Physical obstructions meant to slow down vehicles
	3.4 Bicycle, PTW and pedestrian paths	Dedicated space for cyclists, PTWs and pedestrians to separate them from other motorized traffic	(a) Bicycle lanes (b) PTW lanes (c) Footpaths (d) Footbridges (e) Pedestrian tunnels
	3.5 Traffic control and junctions	All signs, signals, markings, and other devices used to regulate, warn or guide traffic, placed on, over or adjacent to a street, highway, pedestrian facility or bikeway by authority of a public agency having jurisdiction	(a) Road markings and signs (variable signs, zebra crossings, edge line rumble strips, chevrons) (b) Signalized junctions (c) Non‐signalized junctions (d) Roundabouts
	3.6 Work zones and tollbooths		(a) Work zones: A work zone is an area where roadwork takes place and may involve lane closures, detours and moving equipment. (b) Toll roads: The roads for which fee or toll is assessed for passage. The toll is collected by toll booths, plazas etc. (c) Road maintenance: The maintenance of roads to keep them in good condition by carrying out scheduled repairs and reinforcement work
4. Post‐crash pre‐hospital care	4.1 Extrication	Removal of an occupant from a crashed vehicle in case of physical or mechanical entrapment	(a) Road and helicopter ambulances including medical equipment in the ambulance (b) (ALS/ATLS) (c) (BLS/PHTLS)
	4.2 Ambulances	A vehicle equipped to take injured/sick people to and from hospital in case of emergencies	
	4.3 Level of medical personnel	The level of training of medical personnel to manage for acute trauma case	
	4.4 Time to hospital	Time taken to transport the patient from incident site to hospital	
	4.5 Drugs and medications	The availability of appropriate drugs and medications to successfully manage post‐crash emergencies	
	4.6 First aid training of bystanders, drivers and policeman	Assistance and execution of medical aid by onlookers to diminish mortality and damage seriousness	
	4.7 Stay and play versus scoop and run	In case of stay and play patient receives treatment on scene before being transported to hospital while scoop and run means transporting the patient to hospital without any intervention	
5. Legal and Institutional framework	5.1 Pricing		(a) Vehicle taxes: Taxes imposed on vehicles based on engine capacity, sitting capacity, cost price etc. (b) Road pricing: The charges levied on road users to discourage use of certain vehicles, fuel sources or use of busy roads at certain times. (c) Congestion Pricing: A system of surcharging road users that are subject to congestion through excess demand during peak hours (d) Fuel pricing: Change in fuel prices
	5.2 Safety Institutions		(a) Central agencies (b) Research institutions
	5.3 Motor Vehicle Insurance	The insurance schemes for motor vehicles serve dual purpose. First, it is designed to protect individual from major financial losses caused by traffic accidents. Second, these schemes should be designed so that they encourage crash‐free driving, the purchase of safer vehicles and the use of safety equipment for vehicles	
	5.4 Laws	Laws regulating road user behaviour such as use of helmets, seat belts, cell phone, etc.	

#### 3.2.2 Outcomes

The outcomes are listed in outcome domains and each domain has a number of sub‐domains (Table 2). These may be modified during the piloting stage.

**Table 2:** Evidence and gap map outcomes

Domain	Subdomain
Primary outcomes—Health and traffic crashes	1. Fatal crashes 2. Non‐fatal injury crashes
Intermediate outcomes—Safe road‐use practices	1. Change in use of seat belts 2. Change in use of helmets 3. Change in speed 4. Change in drug/alcohol use

We will be excluding any study that includes only vehicular (including bicycles) damage/crash but do not report any related fatalities and injuries.

### 3.3 Criteria for including and excluding studies

#### 3.3.1 Types of study designs

The EGM will include impact evaluations and systematic reviews of the effectiveness of interventions. Impact evaluations are defined as intervention evaluations or field experiments that use quantitative approaches applied to experimental or observational data to measure the effect of an intervention relative to a counterfactual representing what would have happened to the same group in absence of that intervention. Impact evaluations may also test different intervention designs. We will include both completed and on‐going impact evaluations and systematic reviews; to capture the latter, we will include prospective study records in trial registries or protocols when available.

Details of study designs:

(a) Prospective studies allocating the participants to the intervention using randomized or quasi‐randomized mechanisms at individual or cluster levels.
  i. Randomized control trial (RCT) with assignment at individual or cluster level (e.g., clustering at market, round‐about, etc.).
  ii. Quasi‐RCT using a quasi‐random method of prospective assignment (e.g., alternation of clusters).
(b) Non‐randomized designs with selection on unobservables:
  i. Natural experiments using methods such as regression discontinuity (RD).
  ii. Panel data or pseudo‐panels with analysis to account for time‐invariant unobservables (e.g. difference‐in‐difference (DID), or fixed‐ or random‐effects models).
  iii. Cross‐sectional studies using multi‐stage or multivariate approaches to account for unobservables (e.g. instrumental variable, IV, or Heckman two‐step estimation approaches).
(c) Nonrandomized designs with selection on observables:
  i. Controlled before and after studies with an intervention and comparison group using methods to match individuals and groups statistically (e.g., PSM) or control for observable confounding in adjusted regression.
(d) Studies explicitly described as systematic reviews and that describe methods used for search, data collection, and synthesis.

We will include impact evaluations where the comparison/control group receive no intervention (standard road safety intervention), a different intervention (e.g. police enforcement), a placebo or the study employs a pipeline (wait‐list) approach.

All theoretical, modelling or laboratory studies would be excluded. Additionally, all studies using self‐reporting and simulations will also be excluded.

#### 3.3.2 Treatment of qualitative research

We do not plan to include qualitative research.

#### 3.3.3 Types of settings

We will include all studies conducted of interventions aiming to have an impact on traffic crashes. We will exclude studies focusing on car design effectiveness in road safety the reasons for which are explained in scope of the EGM.

#### 3.3.4 Status of studies

We will search for and include completed primary studies and systematic reviews and on‐going systematic reviews. We will not exclude any studies based on language or publication status or publication date.

### 3.4 Search strategy and status of studies

The search strategy will be to cover all the electronic searches possible including thorough searches for both published and “grey” literature. The following strategies will be used to identify completed and ongoing studies:

(1) English Language Database and trial registries: We will be searching SafetyLit (https://www.safetylit.org/), PubMed (https://www.ncbi.nlm.nih.gov/pubmed/), TRID (https://trid.trb.org/), Web of Science database, EMBASE, Cochrane Injuries Group's Specialised Register, Cochrane Central Register of Controlled Trials (CENTRAL), TRANSPORT database, Transportation Research Information Services (TRIS), EASTS database, Scopus, Google Scholar.
(2) Non‐English Databases:
(3) Systematic review database: Epistemonikos (https://www.epistemonikos.org/), Cochrane Library, Campbell Library, 3IE, EPPI centre.
(4) Organization and conference searches: We will be searching for literature using online repositories of organizations who are known to produce or keep depositories of effectiveness evaluations of road safety interventions. These include SafetyCube (https://www.roadsafety‐dss.eu/#/), IIHS (http://iihs.co.in/), SWOV Institute For Road Safety Research (https://www.swov.nl/en), World Health Organization websites (WHO), Asian Development Bank, DFID, Centre for Global Development, International Development Research Centre (IDRC), Australian Road Research Board (ARRB): www.arrb.org.au, UN road safety collaborations, Information and Technology Centres for Transport and Infrastructure—CROW (Netherlands): www.crow.nl, Danish Council for Road Safety Research: www.trm.dk/eng/veje/rft, National Highway Traffic Safety Administration(NHTSA), USA: www.nhtsa.dot.gov, Swedish National Roads Administration: www.vv.se/for_lang/english/, Institute of Transport Economics (TOI), Norway: www.toi.no, Transportation Research Board (TRB): www.nas.edu/trb/, Transport Research Laboratory (TRL),UK: www.trl.co.uk, VTI Swedish National Road and Transport Research Institute: www.vti.se, VTT Finland www.vtt.fi/indexe.htm, the Organisation for Economic Co‐operation and Development's (OECD), Joint Transport Research Centre's International Transport Research Documentation (ITRD) database), European Transport Safety Council (ETSC) https://etsc.eu/, World Conference on Transport Research Society (WCTR) https://www.wctrs‐society.com/, International Research Council On Biomechanics Of Injury (IRCOBI) http://ircobi.org/wordpress/proceedings/, East Asian Science, Technology and Society: An International Journal (EASTS) https://read.dukeupress.edu/easts
(5) Bibliographic searches: There are several systematic reviews relevant to road safety in our scope. We will screen these systematic reviews to identify primary studies from them. We will conduct bibliographic back‐referencing of reference lists of all included systematic reviews and primary studies, and citation searches, to identify additional primary studies and systematic reviews.

A sample search string is included as Appendix B.

### 3.5 Screening and selection of studies

The screening tool (see Appendix D) is based on the inclusion and exclusion criteria described above. Screening will be conducted in two stages: (a) title and abstract, and (b) full text. The reason for exclusion (as per the screening tool) shall be coded at the full text stage.

We will use EPPI Reviewer to assess studies for inclusion at both the title/abstract and full‐text screening stages. Due to time and resource constraints, at the title/abstract stage, we will use EPPI Reviewer's machine learning capabilities to prioritize studies in order of likelihood of inclusion. We will screen until we are no longer finding any studies to include (at least 50 studies with 0 includes). Two researchers will screen each title/abstract and each full‐text. Any disagreements on inclusion will be resolved through discussion, and referred to a third party when agreement cannot be reached.

### 3.6 Data extraction, coding and management

The coding form (Appendix D), based on the framework and PICOS, will be used for data extraction. Data extracted from each study will include bibliographic details, intervention types and descriptions, outcome types and descriptions, study design, context/geographical information, details on the comparison group, and on the quality of the implementation, where available.

The coding of systematic reviews will be based on the characteristics of the included studies in the review which meet the PICOS for this map.

This tool will be piloted to ensure consistency in coding and resolve any issues or ambiguities. A single researcher will conduct the data extraction for each study; however, all coders will be trained on the tool before starting and a sample will be double coded to check for consistency.

We will use EPPI Reviewer to extract descriptive data from all studies meeting our inclusion criteria.

### 3.7 Quality appraisal

We will assess risk of bias, study quality or confidence for all included systematic reviews using AMSTAR 2 checklist (Shea et al., 2017). The tool appraises systematic review conduct, analysis and reporting, guiding appraisers towards an overall judgement of low, medium and high confidence in the review findings.

We will not be critically appraising the quality of the included primary studies (impact evaluations), but will collect data on study design. For the purpose of the present map it is not necessary to critically appraise the interventions effectiveness, beyond indicating whether the evidence is from randomized, nonrandomized studies or observational studies as the systematic reviews provide overviews of the body of evidence, including their quality, where they exist. A major purpose of the map is to provide access to the body of work on particular outcomes and interventions to encourage further syntheses of those studies by researchers in road safety sector.

## 4 ANALYSIS AND PRESENTATION

### 4.1 Unit of analyses

The unit of analysis is the paper. Where multiple papers exist on the same study (e.g. a working paper and a published version), the most recent open access version will be included in the evidence map. If the versions report on different outcomes, an older version will be included for the outcomes not covered in later versions.

We shall code where papers come from the same study (i.e. the same data set by the same research team). It is common in public health to publish multiple papers from the same study, reporting different outcomes or different sub‐populations in separate papers.

### 4.2 Planned analyses

The analysis shall present tabulations and graphs by the primary and secondary dimensions, as well as time trends. Evidence gaps shall be identified and discussed.

### 4.3 Presentation

The EGM will have two primary dimensions: interventions (rows) and outcomes (columns). Additional dimensions will be:

1. Income: Low‐income countries, lower‐middle income countries, upper‐middle income countries, high‐income countries (according to World bank 2020 fiscal year classification).
2. Region: South Asia, Sub‐Saharan Africa, East Asia and Pacific, Europe and Central Asia, Latin America and Caribbean, Middle East and North Africa, North America (according to World bank classification).
3. Systematic review quality: low, moderate, high.
4. Type of primary study: RCT, non‐RCT, Observational study.
5. Status of study (completed, ongoing).

In the hard copy of the EGM, multiple 2 × 2 representations of the EGM will be reported. In the online version, selected additional dimensions will be possible to use as a filter (Appendix A). The online version will include references to included studies and brief summaries of each study (for impact evaluation studies) or plain language summary (for systematic reviews) provided for it.

## 5 STAKEHOLDER ENGAGEMENT

The framework presented here has been developed through the following process:

Stage 1: Initial framework to be constructed through review of strategy and policy documents, and discussions through external consultations through (a) consultation with Advisory Group specially nominated for this project and (b) discussion with ICoRSI Council Members, (c) reference from *The Handbook of Road Safety Measures* (Elvik and Vaa, 2004).

Stage 2: Piloting framework with 30 included studies. The framework will be finalized once the first 30 studies are coded. The protocol will be revised at this point.

## ROLES AND RESPONSIBILITIES

- Content: Dinesh Mohan, Geetam Tiwari, Kavi Bhalla, and Mathew Varghese are members of ICoRSI and have been working on transport research and road safety interventions for many years. These authors will be providing the content expertise for the EGM.
- EGM methods: Howard White as CEO provides technical and strategic support for the development of EGM in Campbell library. Previously, he has initiation and led the development of EGM during his association with 3ie. Denny John is currently co‐author of 3 ongoing EGM registered with Campbell library. Ashrita Saran was lead author of a review of evidence maps, and is leading several on‐going maps, as well as conducting many training workshops on constructing evidence maps.
- Information retrieval: Denny John, and Ashrita Saran, in consultation with Howard White, and other authors will provide information retrieval expertise for the EGM.

## SOURCES OF SUPPORT

This EGM is supported by ICoRSI (Independent Council for Road Safety International).

## DECLARATIONS OF INTEREST

No conflicts of interest.

## PRELIMINARY TIMEFRAME

Approximate date for submission of the EGM: November 2019

## PLANS FOR UPDATING THE EGM

We plan to update the map (or support others in doing so) when sufficient further studies and resources become available.

## Footnotes

^1^
Retrieved from https://www.roadsafety‐dss.eu/#/.

^2^
For more information, see: http://www.3ieimpact.org.en/evidence/gap‐maps/

^3^
INDEPENDENT COUNCIL FOR ROAD SAFETY INTERNATIONAL (for more information refer https://www.icorsi.org/).

## APPENDIX A EVIDENCE MATRIX

**Table:**

**Interventions**	
Human factors	1. Enforcement
	2. Sanctions and Penalties
	3. Driver Training and Licensing
	4. Road user education, awareness building and public campaigns
Vehicle factors and protective devices	1. Bicycle
	2. Powered Two‐wheeler
	3. Bus
	4. Truck
	5. Other Vehicles (excluding Car)
Road design, infrastructure and traffic control	1. Road design and urban form
	2. Protective Infrastructure
	3. Speed Control Infrastructure
	4. Bicycle, PTW and Pedestrian Paths
	5. Traffic Control and Junctions
	6. Work zones and tollbooths
Post‐crash pre‐ hospital care	1. Extrication
	2. Ambulances
	3. Level of medical personnel
	4. First aid training of bystanders
	5. Stay and play vs Scoop and run
	6. Time to hospital
	7. Drugs and medications
Legal and institutional framework	1. Pricing
	2. Safety Institutions
	3. Motor Vehicle Insurance
	4. Laws
**Outcomes**	
Primary Outcomes	1. Fatal crashes
	2. Non‐fatal injury crashes
	3. Property damage only crashes
Intermediate Outcomes	4. Change in use of seat belts
	5. Change in use of helmets
	6. Change in Speed
	7. Change in Alcohol/drug use
**Filters**	
AGE	‐ All Age
	‐ 0‐5 years
	‐ 6 years ‐Licensing Age
	‐ Licensing Age – 65 years
	‐ >65 years
ROAD TYPE	‐ All Road Types
	‐ Urban open access roads
	‐ Urban restricted access roads
	‐ Rural open access roads
	‐ Rural restricted access roads

## APPENDIX B SEARCH TERMS

### Intervention terms for PubMed

**Table:**

Search	Query
#1	Search "Accidents, Traffic"[Mesh] OR "traffic accident*" OR "traffic crash*" OR "road accident*" OR "road crash*" Field: Title/Abstract
#2	Search (("Accidents, Traffic"[Mesh]) AND (#4 OR #5 OR #6 OR #7 OR #8 OR #9 OR #10 OR #11 OR #12 OR #13 OR #14 OR #15 OR #16 OR #17 OR #18 OR #19 OR #20 OR #21 OR #22 OR #23 OR #24 OR #25 OR #26 OR #27 OR #28 OR #29 OR #30 OR #31 OR #32 OR #33 OR #34 OR #35 OR #36 OR #37 OR #38 OR #39 OR #40 OR #41 OR #42 OR #43 OR #44 OR #45 OR #46 OR #47 OR #48 OR #49 OR #50 OR #51 OR #52 OR #53 OR #54)) Field: Title/Abstract
#3	Search pric* AND (road or street or congestion) Field: Title/Abstract
#4	Search "vehicle tax*" Field: Title/Abstract
#5	Search "hospital" AND (crash OR road OR street) Field: Title/Abstract
#6	Search ("first aid" OR "first‐aid") AND (road OR street OR crash) Field: Title/Abstract
#7	Search helicopter AND (road OR street OR crash) Field: Title/Abstract
#8	Search ambulance AND (road OR street OR crash) Field: Title/Abstract
#9	Search extraction AND (crash* OR accident*) Field: Title/Abstract
#10	Search "insurance polic*" AND (crash OR road OR street) Field: Title/Abstract
#11	Search motorcycl* Field: Title/Abstract
#12	Search "median" AND (road OR street) Field: Title/Abstract
#13	Search Shoulder AND (road OR street) Field: Title/Abstract
#14	Search "cycl* lane" OR "bicycl* lane" OR "motorcycl* lane" Field: Title/Abstract
#15	Search (safety organization* OR safety institution*) AND (road or street or crash or accident) Field: Title/Abstract
#16	Search speed camera* Field: Title/Abstract
#17	Search random breath test* Field: Title/Abstract
#18	Search commercial driver* Field: Title/Abstract
#19	Search professional driver* Field: Title/Abstract
#20	Search ("safety in number*" OR "safety‐in‐number") AND (road OR street OR crash) Field: Title/Abstract
#21	Search "traffic volume" Field: Title/Abstract
#22	Search penalty* AND (road OR safety OR crash OR accident) Field: Title/Abstract
#23	Search "license suspen*" AND (road OR safety OR crash OR accident) Field: Title/Abstract
#24	Search (prison or jail) AND (road OR safety OR crash OR accident) Field: Title/Abstract
#25	Search speed* AND (road OR safety OR crash OR accident) Field: Title/Abstract
#26	Search child* AND (Road OR street OR crash OR accident) Field: Title/Abstract
#27	Search "modal share" OR "percentage share" Field: Title/Abstract
#28	Search "traffic calming" Field: Title/Abstract
#29	Search "seat belt*" OR helmet* Field: Title/Abstract
#30	Search bicyclist* OR cyclist* Field: Title/Abstract
#31	Search "driv* under influence" OR "DUI" Field: Title/Abstract
#32	Search "powered two‐wheeler*" OR "PTW" Field: Title/Abstract
#33	Search "driv* license*" Field: Title/Abstract
#34	Search education AND (road or street) Field: Title/Abstract
#35	Search elder* AND (road or street) Field: Title/Abstract
#36	Search campaign* AND (road or street) Field: Title/Abstract
#37	Search "Non motorized vehicle*" OR "NMV" Field: Title/Abstract
#38	Search occupant AND (road OR street) Field: Title/Abstract
#39	Search pedestrian* Field: Title/Abstract
#40	Search "urban road*" OR "rural road" Field: Title/Abstract
#41	Search lighting* AND (road OR street) Field: Title/Abstract
#42	Search (junction* OR intersection*) AND (road OR street) Field: Title/Abstract
#43	Search roundabout* OR rotary Field: Title/Abstract
#44	Search "traffic sign*" Field: Title/Abstract
#45	Search "speed limit*" Field: Title/Abstract
#46	Search "public Transport" Field: Title/Abstract
#47	Search Protect* cloth* AND (road OR street) Field: Title/Abstract
#48	Search "guard rail*" Field: Title/Abstract
#49	Search "lane* width" Field: Title/Abstract
#50	Search "road marking*" Field: Title/Abstract
#51	Search "Stay and play" OR "Scoop and run" Field: Title/Abstract
#52	Search "police enforcement" AND (road OR safety OR crash OR accident) Field: Title/Abstract
#53	Search "work zone*" AND (road OR street) Field: Title/Abstract

## APPENDIX C DATA EXTRACTION TEMPLATE

**Table:**

Number	ID	Description	Question	Coding
**1. Publication details**				
1.01	ID	Unique study identifier	Surname of first author and year of publication	Author ##
1.02	AUTHOR	First author	Surname, initial	Surname, initial
1.03	TITLE	Full title	Full title of paper	Open answer
1.04	SHORT_TITLE	Short title	Short version identifying paper	Open answer
1.05	DATE	Publication date	Year	YYYY
1.06	COUNTRY			Open answer
1.07	LOCATION			Open answer
1.08	REGION	Region of study	Which region was the study conducted in?	1. South Asia 2. Sub‐Saharan Africa 3. East Asia and Pacific 4. Europe and Central Asia 5. Latin America and the Caribbean 6. Middle East and North Africa 7. North America
**2. Intervention details**				
2.01	Human Factors: Any factor contributing to the occurrence and consequences of a crash or affects the behaviour of a road user			1.1 Enforcement
				1.2 Sanctions and Penalties
				1.3 Driver Training and Licensing
				1.4 Road user education, awareness building and public campaigns
2.02	VEHICLE FACTORS AND PROTECTIVE DEVICES: Different vehicles have different characteristics (size, weight….) and has different uses, hence, this intervention includes the effectiveness of vehicle factors and protective equipment (helmet, seatbelt…) in mitigating injuries in road crashes			1.1 Bicycle
				1.2 Powered Two‐wheeler
				1.3 Bus
				1.4 Truck
				1.5 Other Vehicles (excluding Car)
2.03	ROAD DESIGN, INFRASTRUCTURE AND TRAFFIC CONTROL: The design of different road infrastructure aiming to contribute significantly to traffic safety by reducing the number of crashes and crash severity.			1.1 Road design and urban form
				1.2 Protective Infrastructure
				1.3 Speed Control Infrastructure
				1.4 Bicycle, PTW and Pedestrian Paths
				1.5 Traffic Control and Junctions
				1.6 Work zones and tollbooths
2.04	POST‐CRASH PRE‐HOSPITAL CARE: The aim of post‐crash care is to avoid preventable death and disability, limit the severity of the injury and the suffering caused by it, and ensure the crash survivor's best possible recovery and reintegration into society. The way in which persons injured in road traffic crashes are dealt with following a crash determines their chances and the quality of survival			1.1 Extrication
				1.2 Ambulances (Road and helicopter) (including equipment)
				1.3 Level of medical personnel
				1.4 Drugs and medications
				1.5 First‐aid training of bystanders
				1.6 Stay and play vs Scoop and run
				1.7 Time to hospital
2.05	LEGAL AND INSTITUTIONAL FRAMEWORK: The safety laws and regulations play a significant role in reducing injuries and fatalities among all road users. The safety institutions are responsible for the enforcement of these laws, hence their structure influences safety and road‐user behaviour			1.1 Pricing
				1.2 Safety institutions
				1.3 Motor Vehicle Insurance
				1.4 Laws
**3. Study design and data collection**				
3.01	STUDY_DESIGN	Design type	Categorize the study design	1.1 Randomized Control Trial (RCT)
				1.2 Regression Discontinuity
				1.3 Difference‐in‐difference
				1.4 Propensity Score Matching
				1.5 IV/Heckman two‐step
				1.6 Controlled Before‐After
				1.7 Time Series with control group
				1.8 Case‐control
3.02	STUDY_METHODS	Methods of inference	What methods are used to identify the treatment effect?	1.1 DID
				1.2 PSM
				1.3 IV/Heckman selection
				1.4 Multivariate regression/covariates adjusted analysis (e.g. ANOVA analysis)
				1.5 Bivariate analysis/comparison of means
**4. Impacts**				
4.01	IMPACT‐VARIABLE	Impact or final outcome	What impacts are reported (code multiple outcomes in additional columns)?	1.1 Fatal crashes
				1.2 Non‐ Fatal injury crashes
				1.3 Property Damage Only crashes
			1. Health and traffic crashes	2.1 Change in seat‐belt use
			2. Safe road‐use practices	2.2 Change in helmet use
				2.3 Change in speed
				2.4 Change in Alcohol/Drug Use

## APPENDIX D CODING CATEGORIES

**Table:**

Category		Answer
Descriptive information	Title	Open answer
	Author citation	Open answer
	Publication Date	Open answer
	URL	Open answer
	Volume no	Open answer
	Issue no	Open answer
Geographical information	World Bank region	1. South Asia 2. Sub‐Saharan Africa 3. East Asia and Pacific 4. Europe and Central Asia 5. Latin America and Caribbean 6. Middle East and North Africa 7. North America
	Country	1. Low income 2. Lower Middle income 3. Upper Middle income 4. High Income Countries
Study design		1. RCT 2. Regression Discontinuity 3. Difference‐in‐difference 4. Propensity Score Matching 5. IV/Heckman two‐step 6. Controlled Before‐After 7. Time Series with control group 8. Case‐control
Population		ROAD USER
		1. Pedestrian 2. Driver 3. Passenger
		VEHICLE
		1. Powered two‐wheeler (PTW) 2. Car 3. Bus 4. Truck 5. Other Motorized vehicles 6. All Motorized vehicles 7. Bicycle 8. Other Non‐Motorized vehicles
Filters		AGE
		1. All age 2. 0‐5 years 3. years ‐Licensing Age 4. Licensing Age – 65 years 5. > 65 years
		ROAD TYPE
		1. All Road Types 2. Urban open access roads 3. Urban restricted access roads 4. Rural open access roads 5. Rural restricted access roads
Intervention		Human Factors
		1. Enforcement 2. Sanctions and Penalties 3. Driver Training and Licensing 4. Road user education, awareness building and public campaigns
		Vehicle Factors and Protective Devices
		1. Bicycle 2. Powered Two‐wheeler 3. Bus 4. Truck 5. Other Vehicles (excluding Car)
		Road design, Infrastructure and traffic control
		1. Road design and urban form 2. Protective Infrastructure 3. Speed Control Infrastructure 4. Bicycle, PTW and Pedestrian Paths 5. Traffic Control and Junctions 6. Work zones and tollbooths
		Post‐crash pre‐hospital care
		1. Extrication 2. Ambulances (Road and helicopter) (including equipment) 3. Level of medical personnel 4. First aid training of bystanders 5. Stay and play vs Scoop and run 6. Time to hospital 7. Drugs and medications
		Legal and institutional framework
		1. Pricing 2. Safety Institutions 3. Motor Vehicle Insurance 4. Laws
Outcome		Primary Outcome
		1. Fatal crashes 2. Non‐fatal injury crashes 3. Property damage only crashes
		Intermediate Outcome
		1. Change in use of seat belts 2. Change in use of helmets 3. Change in Speed 4. Change in Alcohol/drug use
